# Misdiagnosed Mania Revealing Underlying Paraphilic Disorders: A Case Report

**DOI:** 10.1192/j.eurpsy.2026.12111

**Published:** 2026-07-31

**Authors:** M. Mouldi, I. Mosbah, H. Ghabi, M. Karoui, H. Nefzi, R. Kamoun, F. Ellouze

**Affiliations:** Department of Psychiatry G, Razi Hospital, Manouba, Tunisia

## Abstract

**Introduction:**

Paraphilic disorders involve recurrent, intense sexual urges or behaviors toward atypical objects or situations, causing distress or risk to others. Distinguishing them from benign interests or manic sexual disinhibition is often difficult, as both may present with similar symptoms. Misidentifying paraphilic behaviors as mania can lead to major therapeutic errors due to differing treatment approaches.

**Objectives:**

To illustrate the diagnostic challenges in differentiating paraphilic disorders from manic episodes through a case of multiple paraphilic behaviors initially misdiagnosed as bipolar mania, and to highlight the clinical, therapeutic, and risk-management implications of accurate identification and integrated treatment of multiplex paraphilias.

**Methods:**

This study adopts a descriptive single-case design focusing on a 36-year-old male initially misdiagnosed with bipolar mania. Data were collected through clinical interviews, collateral information, and behavioral observation. Diagnostic clarification was guided by DSM-5 criteria and the Young Mania Rating Scale (YMRS), alongside structured risk assessment tools (Risk Matrix 2000/SV, Static-99R). Comorbidities were evaluated using the PHQ-9 and GAD-7. Management combined fluoxetine therapy and cognitive-behavioral interventions.

**Results:**

Reevaluation showed no manic signs but persistent voyeuristic, exhibitionistic, and gerontophilic behaviors, confirming multiple paraphilic disorders. Risk assessments indicated average recidivism. After switching to fluoxetine and initiating cognitive-behavioral therapy, sexual urges decreased and insight improved. This case shows the risk of confusing paraphilias with mania and stresses the value of tailored, multidisciplinary management with pharmacotherapy, psychotherapy, and risk control.

**Conclusions:**

Multiplex paraphilic disorders may resemble affective syndromes when sexual disinhibition predominates. Accurate differential diagnosis is essential to avoid mismanagement. A comprehensive approach combining pharmacotherapy, psychotherapy, and structured risk monitoring ensures better symptom control, reduced relapse risk, and improved patient and public safety.

**Disclosure of Interest:**

None Declared

